# Effect of Dietary Supplementation of *Durvillaea Antarctica* Meal on Production and Meat Quality Traits of Lambs

**DOI:** 10.3390/ani15020206

**Published:** 2025-01-14

**Authors:** John Quiñones, Rodrigo Huaquipán, Rommy Díaz, Isabela Pérez Núñez, Matías Cortes, Ailín Martínez, Gastón Sepúlveda, Lidiana Velaszquez, David Cancino, Erwin Paz, Néstor Sepulveda

**Affiliations:** 1 Facultad de Ciencias Agropecuarias y Medioambiente, Universidad de La Frontera, Temuco 4780000, Chile; rommy.diaz@ufrontera.cl (R.D.); l.velazquez01@ufromail.cl (L.V.); david.cancino@ufrontera.cl (D.C.); nestor.sepulveda@ufrontera.cl (N.S.); 2 Centro de Tecnología e Innovación de la Carne, Universidad de La Frontera, Temuco 4780000, Chile; r.huaiquipan01@ufromail.cl (R.H.); i.perez04@ufromail.cl (I.P.N.); m.cortes10@ufromail.cl (M.C.); a.martinez26@ufromail.cl (A.M.); g.sepulveda10@ufromail.cl (G.S.); 3 Programa de Doctorado en Ciencias Agroalimentarias y Medioambiente, Universidad de La Frontera, Temuco 4780000, Chile; 4 Carrera de Biotecnología, Universidad de La Frontera, Temuco 4811230, Chile; 5 Programa de Doctorado en Ciencias Mención Biología Celular y Molecular Aplicada, Universidad de La Frontera, Temuco 4811230, Chile; 6 UWA Institute of Agriculture, The University of Western Australia, Perth 6009, Australia; erwin.pazmunoz@uwa.edu.au

**Keywords:** alternative feed, cochayuyo, sheep diet, lamb production, meat quality traits

## Abstract

Worldwide, there are reports indicating that sheep raised in insular systems spontaneously consume seaweed. In the southern hemisphere, there exists *Durvillaea antarctica*, a brown seaweed that possesses minerals and fatty acids that could improve some aspects of sheep production and meat quality, respectively. However, the consumption of this algae in lambs has been scarcely studied. The objective of this study was to evaluate the effects of dietary inclusion of *Durvillaea antarctica* meal on the growth performance, blood profile, and meat quality of fattening lambs. Thirty Araucana Creole lambs were housed and allocated to three pens. One pen served as a control, while the remaining two were supplemented with diets containing 5% and 10% *Durvillaea antarctica* meal. After 9 weeks, the animals were slaughtered. The dietary treatments did not significantly affect body weight and blood biochemical parameters. However, changes were observed in meat quality traits, including increased redness and reduced luminosity in the loin for the high inclusion treatment, in addition to slight alterations in pH and lower lipid oxidation in lambs’ meat fed *Durvillaea antarctica*. Furthermore, the meat from lambs supplemented with *Durvillaea antarctica* exhibited increased levels of linoleic acid and arachidonic acid, along with higher monounsaturated fatty acid content and a reduced omega-3/omega-6 ratio. This study shows that *Durvillaea antarctica* can be used to feed lambs without impairing growth or production parameters, which has been little studied. It is possible that this brown seaweed could be considered a natural additive to improve the quality and nutritional value of lamb meat. The effect of this seaweed on other ruminant models could be addressed in future studies.

## Simple Summary

In some island regions, there are breeds of sheep that feed on seaweed as part of their diet. Seaweed is a feed that has attracted the attention of researchers worldwide due to its high nutritional value. We investigated the effects of feeding a group of lambs with brown seaweed and the impact of this diet on their health status and meat quality. We observed that, in general, 10% seaweed in the diet can improve certain aspects of meat quality such as color, stability, and lipid profile, without negatively affecting the animals’ productive parameters.

## 1. Introduction

The global demand for animal protein is expected to increase significantly by 2050, driven by population growth and dietary changes in developing countries [1,2]. Global meat production has increased more than fourfold since 1961, currently exceeding 350 million tons annually [3]. The FAO projects that global livestock populations will need to increase by 46% by 2050 compared to 2012 levels to meet this growing demand. Current production systems are unlikely to sustain this demand, making it imperative to adopt sustainable and eco-friendly strategies that involve higher levels of intensification [4].

Meat is considered an essential component of a balanced diet, providing proteins, essential amino acids, vitamin B12, and minerals such as zinc, phosphorus, and iron [5], thus representing a product with high nutritional value for the consumer [6]. Despite these nutritional benefits, concerns exist about the high fat content in meat, which is often linked to cardiovascular diseases [7].

Lamb meat, like other ruminant meats, is characterized by high levels of saturated fatty acids (SFAs) and low levels of polyunsaturated fatty acids (PUFAs) [8,9]. Red meat is mainly produced by ruminants such as bovine and ovine species, the latter being a model of great importance due to their size, temperament, adaptability, and tolerance to stress conditions. In response to health-conscious trends, recent research has focused on developing healthier meat products by improving their lipid content and fatty acid profiles [10,11]. Strategies include modifying carcass composition through diet, reformulating products, and optimizing processing conditions [10]. Additionally, studies have explored alternative raw materials for ruminant feed, such as fruit species, agricultural waste, and seaweed, demonstrating that their dietary inclusion does not negatively affect growth, health, or productive parameters [12,13,14]. In this context, the use of alternative raw materials for livestock feed has garnered scientific interest, with marine products emerging as a promising focus [15]. Marine products, mesopelagic fish, zooplankton, and seaweed offer sustainable and nutritious alternatives for livestock feed [16,17]. The efficiency of meat production systems has been significantly impacted by climate change, as factors such as reduced rainfall, heat waves, and cold stress adversely affect grassland species. These environmental stresses diminish the nutritional quality of forage, subsequently reducing the productive performance of livestock [18]. In this context, marine products improve milk yield, enhance meat quality, and reduce methane emissions [16,17]. Continued research into production and processing technologies is essential to fully realize the potential of such products in addressing feed scarcity and supporting environmental sustainability.

Seaweeds, simple autotrophic organisms used as food since ancient times, are gaining attention due to their nutritional characteristics [19,20,21]. Although limited, studies on the dietary inclusion of seaweeds in ruminants have shown promising results. Research on species such as *Ulva lactuca, Chaetomorpha linum*, and *Ascophyllum nodosum* has demonstrated benefits, including improved lipid profiles, stress resilience, and productive performance, without adverse effects on growth or health [22,23,24,25,26]. Additionally, supplementation with brown seaweed like *Ascophyllum nodosum* has shown positive effects on antioxidant activity and stress markers in lambs [27].

Among these, *Durvillaea antarctica*, a brown seaweed endemic to the Southern Hemisphere’s sub-Antarctic regions, has been studied for its bioactive compounds, with antioxidant, anti-inflammatory, and other potential effects [28,29,30]. In Chile, it has been primarily extracted for its bioactive compounds intended to be used for various industrial applications [31]. Its use in animal feed remains limited, although it presents a promising profile [32]. However, comprehensive research on the effects of *D. antarctica* as a feed ingredient on ruminant performance and meat quality is still lacking.

*Durvillaea antarctica* is a valuable source that does not rely on land or freshwater for cultivation and has mineral pigments, antioxidants, and fatty acids that could improve some aspects of sheep production and meat quality. Therefore, the objective of this study was to evaluate the effects of dietary inclusion of *Durvillaea antarctica* meal on the growth performance, health, and meat quality of fattening lambs.

## 2. Materials and Methods

### 2.1. Animal Management and Experimental Design

This study was approved by the Scientific Ethics Committee of the Universidad de La Frontera, Temuco, Chile (N°021/22). Thirty 3-month-old lambs (weights 19.2 kg ± 0.2) were used. The animals were housed in paddocks with natural grassland and subjected to a 15-day acclimatization period.

Following this, specimens of *Durvillaea antarctica* were commercially acquired and thoroughly cleaned with pressurized water to remove sand and excess salt. The cleaned specimens were subjected to a controlled drying process at 50 °C for 24 h in a drying oven. After drying, the material was cut into 5 cm diameter pieces and subsequently pulverized using a ZM 200 micro mill (RETSCH, Düsseldorf, Germany) equipped with a 0.2 mm sieve. The resulting *D. antarctica* meal was stored at −21 °C until analysis and use in feed formulation. After the acclimating process, the selected lambs were randomly assigned to three modules (10 animals per module) based on the methodology described by Tripathi and Karim [33]. The control group (CTRL) (initial weight = 20.2 ± 1.7 kg) was fed a basal diet, while the low inclusion group (LDA) (initial weight = 20.7 ± 1.6 kg) received the basal diet ± 5% *D. antarctica* meal, and a third group, the high inclusion group (HDA, initial weight = 24 ± 1.9 kg) received the basal diet + 10% *D. antartica* meal, formulated according to the nutritional requirements established by the NRC [34]. The concentrate was offered ad libitum twice daily in feeding troughs (2–3% of each animal’s body weight in feed per day).

The basal diet consisted of oats (*Avena sativa*, 60%), triticale (×Triticosecale, 24%), sweet lupin (*Lupinus albus*, 14%), and a mineral premix (2%). The inclusion of *D. antarctica* meal in the LDA and HDA diets led to adjustments in the ingredient proportions, particularly for triticale (reducing to 18% and 13%, respectively) and sweet lupin (increasing to 15% and 18%, respectively), to maintain nutritional balance. The experimental diets were formulated to be isonitrogenous, containing approximately 15% crude protein (DM basis), with metabolizable energy ranging from 2.95 to 2.66 Mcal/kg DM. The essential amino acid profile included lysine (3.77–4.10% of protein), methionine (1.38–1.58%), threonine (3.01–3.32%), and tryptophan (1.03–1.17%). Detailed information about diet composition is available in Table 1. Throughout the experimental period, weekly monitoring of weight and health status was conducted. The experiment lasted 9 weeks, after which the animals were transported to the local slaughterhouse for processing.

### 2.2. Evaluation of Blood Parameters

After 9 weeks, the animals were slaughtered and pre-mortem blood samples were collected from all animals to analyze the biochemical parameters, including creatinine, triglycerides, lactate, calcium, phosphorus, alkaline phosphatase, aspartate aminotransferase, alanine aminotransferase, gamma-glutamyl transferase, total lactate dehydrogenase, magnesium, and total proteins. Analyses were conducted following standard commercial clinical laboratory protocols.

### 2.3. Slaughter and Carcass Evaluation

After slaughter, the carcasses were transported to the laboratory in a refrigerated truck that maintained a constant temperature of 4 °C. Tissue composition was evaluated following the methodology described by Sañudo et al. [35], using the left shoulder as the reference and determining the percentages of muscle, fat, jbone, and cartilage.

### 2.4. Meat Quality Analysis

The pH was measured 24 h post-mortem in the Longissimus dorsi muscle using a penetration pH meter (IQ150, IQ, Scientific Instruments, Round Rock, TX, USA). Color was evaluated using a colorimeter (CR-10 color reader, Konica Minolta, Tokyo, Japan) after 30 min of air exposure, recording the parameters L* (lightness), a* (redness), and b* (yellowness), following the methodology described by Quiñones et al. [36]

### 2.5. Lipid Oxidation (TBAR)

Thiobarbituric acid reactive substances (TBARSs) were identified following the method of Vyncke [37] with minor modifications. The degree of lipid oxidation was quantified by using a chromogenic compound formed from the reaction between malondialdehyde (MDA) and thiobarbituric acid in an acidic environment. Malondialdehyde is the predominant compound generated by the oxidation of lipids, particularly unsaturated ones.

For this analysis, 5 g of the sample was mixed for 10 min with 15 mL of 7.5% trichloroacetic acid (TCA) containing 0.1% propyl gallate and EDTA. The mixture was filtered, and 5 mL of the filtrate was combined with 5 mL of 0.02 M 2-thiobarbituric acid. The resulting solution was incubated for 40 min in a boiling water bath and then cooled, and its absorbance was measured at 532 nm. TBARS concentrations were determined using a standard curve of 1,1,3,3-tetraethoxypropane (TEP) (Sigma-Aldrich, Burlington, MA, USA), with results expressed in mg of MDA per kg of sample.

### 2.6. Fatty Acid Profile Analysis

Lipids were extracted from previously homogenized samples following the methodology described by Folch et al. [38] Fatty acid methyl esters (FAMEs) were prepared by homogenizing total fat with 1.3 mL of 2N potassium hydroxide in methanol and 800 µL of n-hexane (Merck, Darmstadt, Germany) using a magnetic stirrer for 30 min. The mixture was then allowed to rest for 5 min.

FAMEs were analyzed using a gas chromatograph (Clarus 500, Perkin Elmer, Waltham, MA, USA) equipped with a flame ionization detector (FID) and hydrogen as the carrier gas at 250 °C. Separation was performed on an SP™ 2380 fused silica capillary column (60 m × 0.25 mm × 0.2 μm film thickness) (Supelco, Bellefonte, PA, USA), injecting 1 µL of sample extract with nitrogen as the carrier gas. The initial column temperature was set at 150 °C. After 1 min, the temperature was increased at a rate of 1 °C min^−1^ to 168 °C, maintained for 11 min, and then further increased at 6 °C min^−1^ to 230 °C, where it was held for an additional 8 min. Individual fatty acids were identified using a 37-component FAME standard mix (C4-C24) (Supelco, Bellefonte, PA, USA). All of these procedures were performed according to the methodology of Quiñones et al. [36]

### 2.7. Statistical Analysis

Data were analyzed with R 4.4.2, using a completely randomized design with two experimental modules. Normality and the homogeneity of variance were assessed using Shapiro–Wilk and Levene’s tests, respectively. A one-way ANOVA was employed to analyze productive parameters and carcass characteristics. When significant differences were detected, Tukey’s test was applied for pairwise comparisons. The significance level was set at *p* < 0.05. The results are expressed as the mean ± standard error of the mean.

An ANCOVA test was conducted to evaluate potential effects of initial weight and ADG in the final weight of the lambs. The ANCOVA model included treatment as a fixed effect, with interaction terms between treatment groups in an orthogonal arrangement, so the contrast was calculated to compare (1) the control group with the treatment groups and (2) among the treatments groups (LDA and HDA). Initial weight (kg) and average daily gain (ADG, kg/day) were included as covariates to adjust for baseline differences and dietary performance.

The formula for the ANCOVA used in this study can be expressed as follows:

Y_ij_ = μ + α_i_ + β_1_ X_1_ + β_2_ X_2_ + ϵ_ij_

where:

Y_ij_: the final weight of the j-th lamb in the i-th treatment group (dependent variable).
μ: the overall mean of the final weight.
α_i_: the effect of the i-th treatment group (CTRL, LDA, HDA).
X_1_: initial weight (covariate).
X_2_: average daily gain (ADG, Covariate).
β_1_: the regression coefficient for the initial weight covariate.
β_2_: the regression coefficient for the ADG covariate.
ϵ_ij_: the residual error term for the j-th lamb in the i-th treatment group.

## 3. Results

### 3.1. Effect of Durvillaea Antarctica on Growth and Development Parameters

The experimental results demonstrate no significant differences (*p* > 0.05) in live weights among the treatments, as observed in Table 2. Initial live weights were 20.2 ± 1.7 kg for the control group (CTRL), 20.7 ± 1.6 kg for the low inclusion group (LDA), and 24.0 ± 1.9 kg for the high inclusion group (HDA) (*p* = 0.292). Similarly, final live weights were 30 ± 2 kg (CTRL), 31 ± 1 kg (LDA), and 34 ± 3 kg (HDA) (*p* = 0.6).

Regarding the tissue composition of the left shoulder, no significant differences were observed between treatments. Muscle content was 51.11 ± 2.8% (CTRL), 49.85 ± 3.1% (LDA), and 47.31 ± 1.2% (HDA) (*p* = 0.5). Fat content values were 17.56 ± 5.3% (CTRL), 14.9 ± 0.8% (LDA), and 19.10 ± 0.4% (HDA) (*p* = 0.3). Bone content values were 23.48 ± 1.82% (CTRL), 24.62 ± 1.57% (LDA), and 24.00 ± 1.02% (HDA) (*p* = 0.23). Cartilage content was 7.84 ± 2.6% (CTRL), 10.66 ± 1.79% (LDA), and 9.59 ± 1.98% (HDA) (*p* = 0.4).

The ANCOVA results (Table 3) reveal that the initial and average daily gain significantly (*p* < 0.05) influenced the final weight of lambs, which indicates that both covariables impacted the final weight of the lambs. However, the effect of the treatments was not statistically significant, and neither was the comparison between the control group and the treatment groups, which suggests that supplementation with *Durvillaea antarctica* at low or high doses did not significantly alter the final weight of lambs compared to the control group.

The adjusted mean final weights of lambs by treatment group, as determined using the ANCOVA model, are presented in Table 4.

### 3.2. Blood Chemical Profile

The blood biochemical parameters shown in Table 5 did not show significant differences across treatments. Creatinine levels were 134 (13) µmol/L (CTRL), 132 (24) µmol/L (LDA), and 116 (3) µmol/L (HDA) (*p* = 0.4). Lactate concentrations were 7.22 (2.14) µmol/L (CTRL), 8.89 (1.70) µmol/L (LDA), and 4.88 (1.69) µmol/L (HDA) (*p* = 0.2). Total protein levels were 69.23 (2.02) U/L (CTRL), 70.07 (2.02) U/L (LDA), and 74.63 (0.90) U/L (HDA) (*p* = 0.11). Triglycerides showed a trend toward higher values in LDA (0.49 (0.01) µmol/L) compared to CTRL (0.31 (0.06) µmol/L) and HDA (0.33 (0.09) µmol/L), but this was not statistically significant (*p* = 0.06).

Enzyme activity showed no significant differences between groups. Alkaline phosphatase (ALP) levels were 648 (103) U/L (CTRL), 386 (193) U/L (LDA), and 270 (269) U/L (HDA) (*p* = 0.6). Aspartate aminotransferase (AST) values were 113 (12) U/L (CTRL), 140 (10) U/L (LDA), and 137 (19) U/L (HDA) (*p* = 0.3). Gamma-glutamyl transferase (GGT) activity exhibited variability, with 1723 (220) U/L in HDA compared to 472 (256) U/L (CTRL) and 470 (97) U/L (LDA), but the *p*-value (0.06) did not indicate statistical significance.

Electrolyte levels, including calcium and phosphorus, were comparable across treatments. Calcium levels ranged from 2.61 (0.06) mmol/L (CTRL) to 2.76 (0.07) mmol/L (LDA) and 2.80 (0.16) mmol/L (HDA) (*p* = 0.4). Phosphorus levels were 2.28 (0.33) mmol/L (CTRL), 2.43 (0.15) mmol/L (LDA), and 2.22 (0.09) mmol/L (HDA) (*p* = 0.6).

### 3.3. Meat Quality

#### 3.3.1. pH and Color

The effects of *Durvillaea antarctica* meal supplementation on lamb meat quality traits are presented in Table 6. Meat pH showed significant differences between treatments (*p* = 0.0036), with values of 5.60 ± 0.11 for CTRL, 5.68 ± 0.09 for LDA, and 5.57 ± 0.05 for HDA. Specifically, the pH was significantly higher in LDA compared to CTRL (*p* = 0.0036). Significant differences were observed between LDA (12.41 ± 0.48) and HAD (*p* = 0.002).

Loin color parameters differed significantly among treatments. Redness (a*) was higher in HDA (14.93 ± 0.45) compared to CTRL (13.89 ± 0.37) and LDA (12.41 ± 0.48) (*p* = 0.002). Chroma (c*) was also higher in CTRL (18.29 ± 0.30) and HDA (19.21 ± 0.65) than LDA (16.48 ± 0.45) (*p* = 0.002). Lightness (L*) was lower in HDA (36.67 ± 0.46) than in CTRL (37.77 ± 0.90) and LDA (39.75 ± 0.87) (*p* = 0.023). The hue angle (h*) was significantly reduced in HDA (36.3 ± 0.6) compared to CTRL (40.7 ± 0.9) and LDA (41.2 ± 1.1) (*p* < 0.001).

Lipid oxidation, measured by the quantity of TBARSs (mg MDA/kg), was lower in HDA (2.19 ± 0.26) than in CTRL (3.06 ± 0.17) and LDA (2.58 ± 0.38) (*p* = 0.05). Leg color parameters showed no significant differences among treatments.

#### 3.3.2. Proximal Analysis

Meat calcium content did not show significant differences between groups, with CTRL showing 20.56 mg/100 g, while similar values were observed in LDA (21.46 mg/100 g) and HDA (19.41 mg/100 g) (Table 7). Protein content was consistent across treatments, with values ranging from 17.9 g/100 g to 19.0 g/100 g. Selenium levels were below detection limits across all groups, and sodium content remained stable. Zinc content was numerically lower in HDA (16.23 mg/100 g) compared to CTRL (19.75 mg/100 g) and LDA (21.34 mg/100 g) group (Table 7).

#### 3.3.3. Fatty Acid Profile

The effect of *Durvillaea antarctica* meal supplementation on the fatty acid profile of lamb meat can be observed in Table 8. Saturated fatty acids (SFAs) exhibited significant differences, with myristic acid (C14:0) being lower in LDA (2.30 ± 0.22%) than in CTRL (3.27 ± 0.15%) and HDA (3.68 ± 0.12%) (*p* < 0.001). Palmitic acid (C16:0) was higher in HDA (22.18 ± 0.12%) than in CTRL (20.65 ± 0.27%) and LDA (20.21 ± 0.34%) (*p* < 0.001). Stearic acid (C18:0) was lower in HDA (19.7 ± 0.3%) compared to CTRL (23.0 ± 1.2%) and LDA (23.9 ± 0.7%) (*p* < 0.001).

Monounsaturated fatty acid (MUFA) content increased slightly with *D. antarctica* inclusion. Polyunsaturated fatty acids (PUFAs) remained stable overall, but specific PUFAs such as linoleic acid (C18:2n6c) and arachidonic acid (C20:4n6) were higher in HDA. Omega-3 (N3) content decreased with *D. antarctica* suplementation, while omega-6 (N6) content increased, resulting in a lower N3/N6 ratio in the treated groups.

## 4. Discussion

In this study, we assessed the impact of dietary supplementation with *Durvillaea antarctica* meal on productive performance and meat quality, carefully adhering to recommendations that limit seaweed inclusion to 10% to avoid potential adverse effects on digestibility and animal health [39]. For example, while seaweed meal has been associated with changes in ruminal parameters and neutral detergent fiber digestibility [40,41], no adverse effects on growth were noted in this study. This suggests that optimal inclusion rates are additive-specific, with other algae species tolerating higher levels (e.g., 20% for *Ulva lactuca*, known as “sea lettuce” [22]; 20% for *Ruppia maritima* and *Chaetomorpha linum* [24]) while others require more conservative rates to avoid adverse effects on digestibility and health (up to 2% in *Ascophyllum nodosum* [27]; 0.2% in *Spirulina platensis* [42]). This is possibly because mammals can tolerate a maximum level of polysaccharides that differ in their concentrations according to the type of algae.

The inclusion of *D. antarctica* meal in lamb diets had no significant effect on growth, live weight, or tissue composition (muscle, fat, bone, cartilage), consistent with previous studies on seaweed diets in ruminants [43,44]. This can be attributed to the fact that these findings are based on experimental trials where diets were carefully formulated to achieve a balanced energy and protein content [45]. Furthermore, in this study, the results indicate that *D. antarctica* meal has a neutral effect on metabolism; it was noted that blood biochemical parameters (e.g., liver enzymes, creatinine, minerals) were stable across treatments, with minor, non-significant variations. These results align with prior findings revealing that algae supplementation did not markedly alter health indicators, supporting the safety of *D. antarctica* in lamb diets [46,47,48,49,50,51,52,53,54]. Contrary to what has been reported in other studies, it is possible that the minerals and other nutrients present in the seaweed are taken up in advance by the rumen bacteria, which prevents their absorption into the small intestine and therefore into the bloodstream; this could represent a mechanism of ruminal adaptation due to the 90 days of supplementation of the experimental groups, which should be verified in future studies.

Consistent with other research, dietary supplementation of *Durvillaea antarctica* meal influences lamb meat quality traits [55,56,57]. In this study, meat quality analysis revealed significant effects on pH, the color of the loin, and lipid oxidation. The low inclusion group (LDA) exhibited a higher pH (5.68 ± 0.02), potentially linked to less glycogen activity [58], which may improve meat tenderness and shelf life. In mammals, seaweed consumption may play an important role in regulating insulin levels without altering blood glucose [59]. Post-mortem muscle pH is a critical determinant of lamb meat quality, influencing tenderness and overall eating quality, as emphasized by Hopkins et al. (2011) [60]. Faster pH declines have been associated with enhanced meat palatability, while pH serves as a reliable indicator of shear force.

Although not directly examining pH, research on lamb diets supplemented with *Saccharina latissima* reported reductions in cooking loss and shear force, along with improved color stability and enhanced antioxidant potential [61]. These findings suggest that such dietary interventions might influence muscle metabolism and post-mortem biochemical processes, indirectly impacting pH and overall meat quality.

Meat color plays a critical role in consumer purchase decisions, influenced by both its intensity and any instability or alterations. A previous report indicates that meat color is not affected by algal dietary intake [62]. However, in our study, the high inclusion group (HDA) exhibited improved redness and chroma, indicative of enhanced visual appeal. This may be related to the levels of fucoxanthins, a pigment present in high concentrations in *D. antarctica*, which may have accumulated in the muscle, leading to color variations [63]. It is possible that 10% of dietary *D. antarctica* meal could serve as a natural additive for the development of visually more attractive lamb meat for the consumer. This needs to be further validated in future research. Lipid oxidation decreased with increasing *D. antarctica* levels, suggesting antioxidant properties from bioactive compounds like polyphenols [64]. Lipid oxidation affects meat’s stability and shelf life, leading to spoilage by degrading its quality and sensory attributes. Typically measured via TBARs (malondialdehyde), it is associated with undesirable flavors. Unlike studies where *Asparagopsis taxiformis* increased oxidation, our results showed reduced TBARs with higher *D. antarctica* levels (3.06, 2.58, and 2.19 mg MDA/kg for control, LDA, and HDA, respectively; *p* = 0.050), suggesting antioxidant properties from bioactive compounds like polyphenols or carotenoids present in seaweed [31].

The inclusion of *D. antarctica* meal in lamb diets did not influence the proximate composition of lamb meat. Calcium and iron levels showed no significant differences between the low or high inclusion groups and the control; therefore, the inclusion of seaweed meal does not affect the meat’s nutritional value. On the other hand, selenium content remained very low across all groups, and sodium levels were slightly higher in the low inclusion group. Zinc content was elevated in the low inclusion group but decreased in the high inclusion group. These findings highlight the potential nutritional trade-offs in the inclusion of dietary seaweed in lambs’ diets, which should be considered when evaluating its impact on lamb meat’s nutritional quality, warranting further exploration of the underlying mechanisms and implications for consumer health. This is consistent with previously reported results in pigs, where dietary *Chlorella vulgaris* increased potassium levels but decreased calcium and manganese [65], and in dairy bovines, where *Spirulina platensis* supplementation did not significantly alter milk or blood mineral concentrations, although it increased fecal mineral content [66]. Despite this, other investigations in lamb show that algae meal supplementation does not negatively impact carcass traits or meat quality parameters [67]. It should also be considered that rumen microbiota can significantly influence nutrient utilization and metabolism in ruminants [68] and can be altered by different feeding regimens and dietary supplements, which in turn affects nutrient absorption and utilization [69,70], and potentially mineral absorption as well.

Regarding meat lipid composition, the dietary inclusion of *D. antarctica* meal altered the fatty acid profiles of lamb meat. Previous studies have shown that seaweed supplementation can significantly increase health-beneficial omega-3 fatty acids, particularly EPA and DHA, in lamb meat [45,71,72]; however, in this study, omega-3 fatty acid levels decreased, while omega-6 fatty acids and the omega-6/omega-3 ratio increased, which raises concerns about the nutritional value of the treated meat. These findings contrast with studies where microalgae supplementation enhanced omega-3 content and improved the omega-3/omega-6 ratio. Similar effects were observed with supplementation using *Saccharina latissima*, likely due to differences in species and inclusion levels [62,73]. Sustainable harvesting practices and optimized formulations are crucial to maximizing the benefits of *D. antarctica* while maintaining both environmental sustainability and nutritional balance [74,75,76].

In this study, linoleic acid (C18:2n6c), a major omega-6 fatty acid, increased notably in the high inclusion group (HDA), with smaller increases in the LDA group. This suggests that *D. antarctica* serves as a source of omega-6 fatty acids when included in lamb diets. Dietary fatty acids in ruminants undergo a biohydrogenation process in the rumen, which affects their composition and absorption. However, some fatty acids manage to escape this process and are deposited in the tissues. The shift toward a higher omega-6 content alters the omega-3/omega-6 ratio, potentially influencing inflammation and overall health. Additionally, monounsaturated fatty acids (MUFAs) showed a significant increase in both the LDA and HDA groups, indicating that the dietary intervention favored the production of beneficial MUFAs, such as oleic acid, which are known for their positive effects on cardiovascular health. Although the total polyunsaturated fatty acid (PUFA) content did not show significant differences, the composition of PUFAs, particularly the increase in omega-6 fatty acids, could have negative implications for human health and consumption. The omega-3 to omega-6 ratio, a crucial indicator of the potential health effects of dietary fatty acids [77], was significantly reduced in the treated groups, decreasing from 0.62 in the control group to 0.43 and 0.41 in the LDA and HDA groups, respectively. This shift toward a higher omega-6 proportion may diminish the health benefits associated with omega-3 fatty acids, as an elevated omega-6 to omega-3 ratio is generally less favorable for health outcomes. While the inclusion of *D. antarctica* in lamb diets enhances certain aspects of the fatty acid profile, it also reduces omega-3 content and disrupts the omega-3 to omega-6 balance, potentially altering the nutritional quality of the meat. This imbalance is particularly relevant, as a high omega-6/omega-3 ratio has been linked to the pathogenesis of chronic diseases, including cardiovascular disease [78].

Some studies suggest that meat omega-3 fatty acids individually have anti-inflammatory properties, whereas omega-6 fatty acids tend to promote inflammation. This emphasizes the importance of maintaining an optimal balance between these fatty acids for homeostasis and metabolic health [79]. However, it is worth noting that some studies, such as Ibiebele et al. (2012) [80], have reported no significant association between omega-3 intake and certain health outcomes, such as ovarian cancer risk. These findings underscore the need for further research in this area.

On the other hand, other studies suggest that omega-6 fatty acids may regulate antioxidant pathways and modulate inflammatory processes, potentially influencing hepatic lipid metabolism and physiological responses in other organs [81]. Omega-6 fatty acids are essential for various physiological functions; however, their impact on human health—particularly in relation to cardiovascular disease and other health conditions—remains a subject of ongoing debate.

Dietary supplementation with seaweed in cattle generally has a positive effect on some key aspects, such as the production system, due to the reduction in methane gas production, weight gain, the improved mineral status, and the increase in healthier fatty acids in the meat. Some studies even suggest an improvement in the immunological and reproductive status of the animals [82,83]. In this study, we could not address these aspects; however, in a preliminary way, we were able to establish that the dietary supplementation of *Durvillaea antarctica* meal does not negatively influence the productive characteristics of the lamb but apparently influences some parameters of the quality of the meat of this species. This allows us to better understand the metabolism of sheep species and its plasticity and the use of *Durvillaea antarctica* as a dietary supplement in various domestic species, which broadens the value of seaweed in the Southern Hemisphere of our planet.

## 5. Conclusions

In this study, the dietary treatments had no significant effect on body weight or blood biochemistry, but influenced meat quality, with increased redness and reduced luminosity of the loin, slight changes in pH, and reduced lipid oxidation in meat from lambs fed *Durvillaea antarctica*. In addition, this diet increased the levels of linoleic and arachidonic acid, monounsaturated fatty acids, and the omega-6/omega-3 ratio. Seaweeds can be offered as a natural dietary supplement to sheep, but the effects vary in relation to the design of the diet and the type of seaweed available for supplementation. Lambs in general represent a source of high-value feed and income for large and small farmers. Therefore, strategies to feed sheep with new raw materials such as *Durvillaea antarctica* must first undergo a cost–benefit analysis and must improve the management systems of this species which is not currently produced artificially. This could endanger the value chain of sheep production and the existence of *D. antarctica*. There are studies developed in humans that indicate that *D. antarctica* could serve as an immunostimulant. Future studies will allow us to address these observations in ruminants, such as the ovine species.

## Figures and Tables

**Table 1:** Nutritional characteristics of experimental lamb diets.

Ingredient Composition	CTRL	LDA	HDA
Oats (*Avena sativa*) (%)	60	60	57
Triticale (× Triticosecale) (%)	24	18	13
Sweet lupin (*Lupinus albus*) (%)	14	15	18
Mineral mix (%)	2	2	2
*Durvillaea antarctica* meal (%)	0	5	10
**Chemical composition**			
Dry matter (% as feed)	86.48	82.14	77.81
Crude protein (% DM)	15.33	15.05	15.40
Metabolizable energy (Mca/kg DM)	2.95	2.80	2.66
Crude fiber (% DM)	12.44	12.44	12.39
**Amino acid profile**			
Lysine (% protein)	4.10	3.93	3.77
Tryptophan (% protein)	1.17	1.11	1.03
Threonine (% protein)	3.32	3.16	3.01
Methionine (% protein)	1.58	1.49	1.38
**Mineral content**			
Calcium (% DM)	1.30	1.3	1.33
Phosphorus (% DM)	3.82	3.64	3.50

DM = dry matter. The control group (CTRL) received the basal diet; the low inclusion group (LDA) received the basal diet ± 5% *D. antarctica* meal; and a third group, the high inclusion group (HAD), received the basal diet + 10% *D. antartica* meal.

**Table 2:** Effect of dietary *Durvillaea antarctica* on growth performance and carcass composition of lambs.

Variable	Value	CTRL ^1^	LDA ^1^	HDA ^1^	*p*-Value ^2^
Weight	Initial weight (kg)	20.2 (1.7)	20.7 (1.6)	24.0 (1.9)	0.252
	Final weight (kg)	30 (2)	31 (2)	34 (3)	0.632
	Average daily gain (kg/d)	0.15 (0.02)	0.13 (0.02)	0.13 (0.02)	0.672
Tissue composition	Bone (%)	23.48 (1.82)	24.62 (1.57)	24.00 (1.02)	0.957
	Cartilage (%)	7.84 (2.60)	10.66 (1.79)	9.59 (1.98)	0.393
	Fat (%)	17.6 (5.3)	14.9 (0.8)	19.1 (0.4)	0.301
	Muscle (%)	51.1 (2.8)	49.9 (3.1)	47.3 (1.2)	0.491

^1^ Mean (std.error). ^2^ ANOVA test. The control group (CTRL) received the basal diet; the low inclusion group (LDA) received the basal diet ± 5% *D. antarctica* meal; and a third group, the high inclusion group (HAD), received the basal diet + 10% *D. antartica* meal.

**Table 3:** Effects of *Durvillaea antarctica* meal supplementation on final weight of lambs, adjusted for initial weight and average daily gain (ADG) with ANCOVA type III.

Variable	Contrast	Estimate (Std. Error)1	Type III Sum Sq	Df	F Value	Pr (>F) 2
(Intercept)		0.37 (3.24)	0.10	1	0.01	0.910
Treatment			10.59	2	0.70	0.510
	CTRL × (LDA/HDA)	0.14 (0.44)				
	LDA × HDA	−0.87 (0.79)				
Initial weight (Kg)		1.15 (0.13) *	583.31	1	77.35	0.000 **
ADG (Kg/day)		45.16 (12.98) *	91.28	1	12.10	0.003 **
Residuals			120.66	16		

Significance codes: <0.05 * and <0.01 **; 1 p was calculated with T-test with the estimate values; 2 p was calculated with F among the covariables. The control group (CTRL) received the basal diet; the low inclusion group (LDA) received the basal diet ± 5% *D. antarctica* meal; and a third group, the high inclusion group (HAD), received the basal diet + 10% *D. antartica* meal.

**Table 4:** Adjusted mean final weights of lambs by treatment group with 95% confidence intervals calculated using the ANCOVA model.

Treatments	Adj. Mean (Std. Error)	CI Lower 95%	CI Upper 95%
CTRL	31.07 (1.01)	28.92	33.23
LDA	32.37 (1.05)	30.14	34.59
HDA	30.62 (1.18)	28.11	33.14

The control group (CTRL) received basal diet; the low inclusion group (LDA) received the basal diet ± 5% *D. antarctica* meal; and a third group, the high inclusion group (HAD), received the basal diet + 10% *D. antartica* meal.

**Table 5:** Effect of dietary *Durvillaea antarctica* meal on biochemical profile of lambs.

Variable	Value	CTRL ^1^	LDA ^1^	HDA ^1^	*p*-Value ^2^
Chemistry	Creatinine (umol/L)	134 (13)	132 (24)	116 (3)	0.430
	Lactate (umol/L)	7.22 (2.14)	8.89 (1.70)	4.88 (1.69)	0.202
	Total proteins (U/L)	69.23 (2.02)	70.07 (2.02)	74.63 (0.90)	0.113
	Triglycerides (umol/L)	0.31 (0.06)	0.49 (0.01)	0.33 (0.09)	0.065
Enzymes	ALP (U/L)	648 (103)	386 (193)	270 (269)	0.587
	AST (U/L)	113 (12)	140 (10)	137 (19)	0.288
	GGT (U/L)	472 (256)	470 (97)	1723 (220)	0.061
	LDH (mmol/L)	87 (12)	66 (15)	88 (7)	0.301
	LDL (mmol/L)	1517 (151)	2030 (229)	2234 (240)	0.099
Minerals	Calcium (mmol/L)	2.61 (0.06)	2.76 (0.07)	2.80 (0.16)	0.361
	Phosporous (mmol/L)	2.28 (0.33)	2.43 (0.15)	2.22 (0.09)	0.561

^1^ Mean (std.error). ^2^ Kruskal–Wallis rank sum test. The control group (CTRL) received the basal diet; the low inclusion group (LDA) received the basal diet ± 5% *D. antarctica* meal; and a third group, the high inclusion group (HAD), received the basal diet + 10% *D. antartica* meal.

**Table 6:** Effect of dietary *Durvillaea antarctica* meal on meat quality traits of lambs.

Variable	Value	CTRL ^1^	LDA ^1^	HDA ^1^	*p*-Value ^2^
Loin Color	a*	13.89 (0.37) ^ab^	12.41 (0.48) ^a^	14.93 (0.45) ^b^	0.002
	b*	11.89 (0.16) ^a^	10.77 (0.27) ^b^	11.02 (0.29) ^b^	0.005
	c*	18.29 (0.30) ^a^	16.48 (0.45) ^b^	19.21 (0.65) ^a^	0.002
	L*	37.77 (0.90) ^ab^	39.75 (0.87) ^a^	36.67 (0.46) ^b^	0.023
	h*	40.7 (0.9) ^a^	41.2 (1.1) ^a^	36.3 (0.6) ^b^	<0.001
Leg Color	a*	8.05 (0.44)	9.29 (0.50)	9.05 (0.77)	0.253
	b*	8.23 (0.91)	6.99 (0.69)	7.42 (0.82)	0.719
	c*	11.85 (0.90)	11.79 (0.70)	11.89 (0.97)	0.991
	L*	46.3 (1.3)	43.4 (0.7)	44.6 (1.6)	0.131
	h*	42 (3)	36 (2)	38 (3)	0.416
pH		5.60 (0.03) ^a^	5.68 (0.02)^b^	5.57 (0.01) ^ab^	0.003
TBARS	MDA (mg/Kg)	3.06 (0.17) ^a^	2.58 (0.38) ^ab^	2.19 (0.26) ^b^	0.050

^1^ Mean (std.error). ^2^ Kruskal–Wallis rank sum test. Different letters indicate significant differences between groups (Wilcoxon test *p* < 0.05). The control group (CTRL) received the basal diet; the low inclusion group (LDA) received the basal diet ± 5% *D. antarctica* meal; and a third group, the high inclusion group (HAD), received the basal diet + 10% *D. antartica* meal.

**Table 7:** Proximal composition of meat from lambs fed different levels of *Durvillaea antarctica* meal.

Variable	CTRL ^1^	LDA ^1^	HDA ^1^	*p*-Value ^2^
Calcium (mg/100 g)	20.56 (1)	21.46 (7)	19.41 (1)	0.372
Iron (mg/100 g)	24.34 (2.4)	16.83 (2.2)	20.38 (2.6)	0.692
Protein (g/100 g)	18.1 (0.38)	17.9 (0.36)	19.0 (0.49)	0.278
Selenium (mg/100 g)	<0.04	<0.04	<0.04	0.800
Sodium (mg/100 g)	74.3 (1.9)	78.8 (2.3)	76.3 (2.9)	0.710
Zinc (mg/100 g)	19.75 (0.3)	21.34 (4.9)	16.23 (1.2)	0.117

^1^ Mean (std.error). ^2^ Kruskal–Wallis rank sum test. The control group (CTRL) received the basal diet; the low inclusion group (LDA) received the basal diet ± 5% *D. antarctica* meal; and a third group, the high inclusion group (HAD), received the basal diet + 10% *D. antartica* meal.

**Table 8:** Effect of dietary *Durvillaea antarctica* meal on fatty acid profile of lamb meat.

Fatty Acids (%)	CTRL ^1^	LDA ^1^	HDA ^1^	*p*-Value ^2^
C 14:0	3.27 (0.15) ^a^	2.30 (0.22) ^b^	3.68 (0.12) ^a^	<0.001
C 16:0	20.65 (0.27) ^a^	20.21 (0.34) ^a^	22.18 (0.12) ^b^	<0.001
C 16:1	1.84 (0.11) ^a^	1.21 (0.11) ^b^	1.70 (0.08) ^a^	<0.001
C 17:0	1.75 (0.12) ^a^	1.27 (0.08) ^b^	1.45 (0.06) ^ab^	0.001
C 18:0	23.0 (1.2) ^a^	23.9 (0.7) ^a^	19.7 (0.3) ^b^	<0.001
C 18:1n 9c	39.09 (0.39) ^a^	40.68 (0.21) ^b^	40.24 (0.20) ^b^	<0.001
C 19:0	1.83 (0.06)	1.74 (0.07)	1.91 (0.05)	0.122
C 18:2n 6c	3.61 (0.25) ^a^	3.82 (0.06) ^ab^	4.25 (0.07) ^b^	0.008
C 20:3n 3	1.25 (0.23)	0.94 (0.02)	0.95 (0.03)	0.131
C 20:4n 6	1.76 (0.15) ^a^	2.32 (0.12) ^b^	2.26 (0.03) ^b^	0.001
C 20:5n 3	0.98 (0.08) ^a^	0.70 (0.05) ^b^	0.95 (0.03) ^b^	<0.001
C 22:6n 3	0.99 (0.08) ^a^	0.95 (0.03) ^a^	0.75 (0.03) ^b^	0.003
SFA	50.50 (0.96)	49.38 (0.19)	48.91 (0.29)	0.118
MUFA	4.93(0.59)	41.89 (0.26)	41.93 (0.21) ^●^	0.047
PUFA	8.58 (0.56)	8.73 (0.17)	9.16 (0.13)	0.412
N 3	3.22 (0.29) ^a^	2.59 (0.05) ^b^	2.65 (0.09) ^b^	0.016
N 6	5.36 (0.40) ^a^	6.14 (0.16) ^b^	6.51 (0.05) ^b^	0.004
N 3/N 6	0.62 (0.05) ^a^	0.43 (0.02) ^b^	0.41 (0.01) ^b^	<0.001

^1^ Mean (std.error). ^2^ ANOVA test. Different letters indicate significant differences between groups (*t* test *p* < 0.05). A black circle (^●^) indicates significant differences between the control group and each treatment group (Dunnet test *p* < 0.05). The control group (CTRL) received the basal diet; the low inclusion group (LDA) received the basal diet ± 5% *D. antarctica* meal; and a third group, the high inclusion group (HAD), received the basal diet + 10% *D. antartica meal*. SFA = saturated fatty acid; MUFA = monounsaturated fatty acid; PUFA = polyunsaturated fatty acid.

## Data Availability

All data is contained within the article.
